# Bibliography

**Published:** 1894-03

**Authors:** 


### Bibliography.

The Dental Cosmos Calendar, published by the S. S. White Dental Manufacturing Company.

This excellent arrangement presented to the subscribers of the *Dental Cosmos* is admirably arranged for appointments, but it is also excellent as a daily memorandum for outside engagements. The publishers request suggestions for the betterment of this calendar from dentists. It seems, however, to comprise all that is needed for the objects intended.

Pearson's Dentists' Appointment-Book, published by R. J. Pearson & Co., Kansas City, Mo.

This is a very neat and convenient pocket appointment-book, and would be entirely satisfactory if the publishers would add the date of the month to the days of the week.

